# Frontotemporal disorders: the expansive panoply of syndromes and spectrum of etiologies

**DOI:** 10.3389/fneur.2023.1305071

**Published:** 2024-01-09

**Authors:** Michael Hoffmann, Fabian Rossi, Lourdes Benes Lima, Christian King

**Affiliations:** ^1^University of Central Florida, Orlando, FL, United States; ^2^Roskamp Institute, Sarasota, FL, United States; ^3^Orlando VA Healthcare System, Orlando, FL, United States

**Keywords:** frontotemporal lobe disorders (FTD), etiological categories, veterans, cardiovascular risk factors, behavioral neurological evaluation

## Abstract

**Background:**

Frontotemporal lobe disorders (FTD) are amongst the most common brain neurodegenerative disorders. Their relatively covert, frequently subtle presentations and diverse etiologies, pose major challenges in diagnosis and treatments. Recent studies have yielded insights that the etiology in the majority are due to environmental and sporadic causes, rather than genetic in origin.

**Aims:**

To retrospectively examine the cognitive and behavioral impairments in the veteran population to garner the range of differing syndrome presentations and etiological subcategories with a specific focus on frontotemporal lobe disorders.

**Methodology:**

The design is a retrospective, observational registry, case series with the collection of epidemiological, clinical, cognitive, laboratory and radiological data on people with cognitive and behavioral disorders. Inclusion criteria for entry were veterans evaluated exclusively at Orlando VA Healthcare System, neurology section, receiving a diagnosis of FTD by standard criteria, during the observation period dated from July 2016 to March 2021. Frontotemporal disorders (FTD) were delineated into five clinical 5 subtypes. Demographic, cardiovascular risk factors, cognitive, behavioral neurological, neuroimaging data and presumed etiological categories, were collected for those with a diagnosis of frontotemporal disorder.

**Results:**

Of the 200 patients with FTD, further cognitive, behavioral neurological evaluation with standardized, metric testing was possible in 105 patients. Analysis of the etiological groups revealed significantly different younger age of the traumatic brain injury (TBI) and Gulf War Illness (GWI) veterans who also had higher Montreal Cognitive Assessment (MOCA) scores. The TBI group also had significantly more abnormalities of hypometabolism, noted on the PET brain scans. Behavioral neurological testing was notable for the findings that once a frontotemporal disorder had been diagnosed, the four different etiological groups consistently had abnormal FRSBE scores for the 3 principal frontal presentations of (i) abulia/apathy, (ii) disinhibition, and (iii) executive dysfunction as well as abnormal Frontal Behavioral Inventory (FBI) scores with no significant difference amongst the etiological groups. The most common sub-syndromes associated with frontotemporal syndromes were the Geschwind-Gastaut syndrome (GGS), Klüver-Bucy syndrome (KBS), involuntary emotional expression disorder (IEED), cerebellar cognitive affective syndrome (CCA), traumatic encephalopathy syndrome (TES) and prosopagnosia. Comparisons with the three principal frontal lobe syndrome clusters (abulia, disinhibition, executive dysfunction) revealed a significant association with abnormal disinhibition FRSBE T-scores with the GGS. The regression analysis supported the potential contribution of disinhibition behavior that related to this complex, relatively common behavioral syndrome in this series. The less common subsyndromes in particular, were notable, as they constituted the initial overriding, presenting symptoms and syndromes characterized into 16 separate conditions.

**Conclusion:**

By deconstructing FTD into the multiple sub-syndromes and differing etiologies, this study may provide foundational insights, enabling a more targeted precision medicine approach for future studies, both in treating the sub-syndromes as well as the underlying etiological process.

## Background

Our understanding of neurodegenerative disease and dementia has evolved rapidly in the last few decades. As recently as the 1970s and 1980’s all dementia was generally considered to be Alzheimer’s disease (AD) (1). Frontotemporal lobe disorders (FTD) are now amongst the most common neurodegenerative disorders, after an approximate 130-year diagnostic hiatus, due largely to the under recognition of Pick’s disease, first described in 1896 (2). The most important reason appears to have been that Pick bodies are found in only 20% of FTD. The pathology in frontotemporal lobe dementia is now known to be due to several associated pathologies such as TDP-43 (A, B, C), tau, FUS (3) and less commonly Pick bodies. In addition to the behavioral and primary progressive aphasia subtypes (semantic aphasia, non-fluent aphasia), cortico-basal degeneration, progressive supranuclear palsy and amyotrophic lateral sclerosis are other recognized clinical variants. In a recent wide-ranging retrospective cohort study, the Frontotemporal Dementia Incidence European Research Study (FRONTIERS) in 9 European countries concluded that the annual incidence rate of 2.36 cases per 100,000 person-years appears more common than formerly appreciated and should be considered irrespective of age (4). In contrast to the frontotemporal dementias, recent studies have yielded insights that the majority may be due to environmental and sporadic causes, rather than being genetic in origin and presenting with mild to moderate behavioral impairment termed frontotemporal disorders or syndromes, rather than dementia (5, 6).

From a diagnostic point of view, much depends on what clinical population is being studied. Cognitive and behavioral neurological disorders are common in the Veteran population as sequelae of traumatic brain injury, post-traumatic stress disorders and neurotoxicological exposure, often presenting as frontotemporal syndromes (7–9). Their relatively covert and frequently subtle presentations and diverse etiologies, pose major challenges in diagnosis and treatments. Yet, these conditions often afflict the most plastic areas of the brain providing potential opportunities for successful interventions. Many, widely differing pathophysiological entities for frontotemporal syndromes have been reported. These include traumatic brain injury (10), vascular causes (11), neurotoxicological syndromes such as Gulf War Illness (primarily a synaptopathy, associated with acute phase lipids, Agent Orange exposure) (9, 12), autoimmune disorders (13), cerebral mechanical aberrations such as sagging brain syndromes (14), infectious causes, including Whipple’s disease (15) and traumatic encephalopathy/chronic traumatic encephalopathy spectrum (16), in addition to the group of frontotemporal lobe dementias. Furthermore, milder forms of FTD with little deterioration over time, such as the mild frontotemporal phenocopy variant have been reported (3). Some of these frontotemporal syndromes may stabilize for decades and even improve, in contradistinction to the traditional dementias such as Alzheimer dementia (AD). Hence timeous and precise diagnosis may allow precision treatment strategies to be implemented.

Mild cognitive impairment occurs years to decades prior to AD and importantly, from a clinical perspective, 30% may have a remediable underlying cause and improve with appropriate treatment (17, 18). Mild behavioral impairment, with recent validated scales, is now also being recognized with similar opportunities for intervention (19). Metabolic syndromes which tend to target the more posterior association areas and the default mode network, often result in the Alzheimer’s spectrum of cognitive dysfunction. The more anterior association cortices of the frontal and anterior temporal lobes are more prone to trauma, toxins and stressors of various kinds, target primarily the salience network and present primarily with an array of behavioral neurological syndromes (20).

### Aims

To retrospectively examine the cognitive and behavioral impairments in the veteran population to garner the range of differing syndrome presentations and etiological subcategories with a specific focus on frontotemporal lobe disorders (FTD).

## Methodology

The study design was a retrospective observation case series with analysis of veterans with cognitive and behavioral disorders. FTD were specifically documented, which were encountered exclusively at the Orlando VA Healthcare System (OVAMC), neurology service, Orlando, Florida. The data collection comprised of demographic, epidemiological, clinical, cognitive neurological, behavioral neurological, laboratory and neuroradiological data. The inclusion criteria constituted veterans evaluated exclusively at the OVAMC neurology section, receiving a diagnosis of FTD by standard criteria, during the observation period dated from July 2016 to March 2021. Exclusion criteria consisted of veterans with cognitive and behavioral syndromes not amenable for further analysis, due to significant behavioral obstacles, multiple comorbid medical or psychiatric conditions or unwilling to undergo further testing. The high number of antipsychotic medications in this exclusion group typically masqueraded with frequent and significant frontal lobe syndromes (abulia, dysexecutive syndrome) as recognized side effects of this class of medications. After syndrome analysis, further exclusions included AD, Lewy Body dementia (LBD), vascular dementia, and mixed dementia syndromes. The studies involving humans were approved by Orlando VA Institutional Research Board Approval IRBNET ID: 1256151-4 and a finalized the approval date on April 11, 2020.

**Figure 1:**
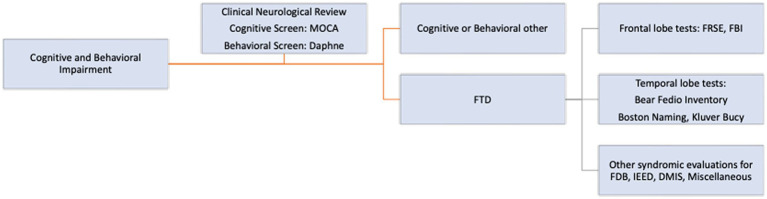
Cognitive and behavioral neurological assessment.

In addition to standard neurological evaluation, a comprehensive screening cognitive and behavioral neurological tool with pre-defined syndromes, according to standard definitions, published elsewhere, was used to guide initial diagnosis (21). Whenever possible the following cognitive and behavioral neurological tests were administered (Figure 1). These included a general cognitive screening test, the MOCA 5-min version (22), a behavioral neurological FTD screening test, the Daphne 6 and 40 (23) and activities of Daily Living using the Katz Disability Scale (24). In addition, specific frontal behavioral tests, the FRSBE (25), Frontal Behavioral Inventory (26) and specific anterior temporal lobe tests and other cognitive syndrome evaluations were used. These included the Boston Naming Test (27), the Bear-Fedio Inventory (BFI) (modified) (28), a Human Klüver Bucy Syndrome Inventory (KBS) (29, 30), a Geschwind-Gastaut inventory (GGS) (31), a delusional misidentification syndrome inventory (DMIS) (32), an involuntary emotional expression disorder (IEED) inventory (33) and a cerebellar cognitive affective syndromes (CCA) inventory (34) (Figure 1). In brief the Human Klüver Bucy syndrome required evidence of any 3 components of (i) visual agnosia, (ii) loss of anger, fear responses with placidity or flattened affect, (iii) altered sexual activity or orientation, (iv) hyperorality or bulimia and hypermetamorphosis (compulsion to manipulate objects in the immediate environment, akin to utilization behavior). The Geschwind-Gastaut syndrome diagnosis was made if any 3 of the following were present; a personality syndrome comprising of: (i) circumstantiality (excessive verbal output, loquacious, hypergraphia, interpersonal viscosity) (ii) Intensified mental life (deepening of emotions, hypermoralism, nascent metaphysical interests, hyper-philosophical), (iii) hyper-religiosity (multiple conversions, deep religious beliefs, mystical states), (iv) altered sexuality (hyposexuality, hyper-sexualism, gender dysphoria, transvestism). DMIS diagnosis was made if a person incorrectly identifies or duplicates persons, places, objects, or even events which may be learned by self-report or substantiated from family members or friends. Many different DMIS have been reported but only 3 types were recorded, including: (i) Capgras syndrome; the belief by the person that a familiar individual or even the person themselves had been replaced by an imposter (hypo-identification), (ii) Fregoli’s syndrome; the belief that an individual familiar to the person is actually impersonating and is presenting themselves as a stranger (hyper-identification) and (iii) intermetamorphosis; two people, both familiar to the person, have interchanged identities with one another. For IEED, item number 6 of FRSBE test was used and was graded on a 5-point Likert scale and if ≥3 was used as positive diagnosis. This delineated a syndrome characterized by spontaneous outbursts of crying, laughing or both, occurring contextually inappropriately. Cerebellar cognitive affective syndromes (CCA) were diagnosed if there was a relevant cerebellar lesion such as stroke or neoplasm with co-occurring onset of cognitive, behavioral or emotional impairment. Traumatic encephalopathy syndrome was diagnosed according to the criteria proposed by the National Comorbidity Survey Replication with at least one of the core criteria and two of the 9 supportive criteria required for diagnosis (35).

Neuroimaging was performed in all patients, including multimodality MRI imaging sequences (GE 3 Tesla) MRI (T1, T2), fluid attenuation inversion recovery (FLAIR) and diffusion weighted imaging (DWI). CT brain scans were a surrogate if MRI contra-indicated and PET brain (FDG, metabolic) scans were performed in selected patients.

Laboratory testing included routine cognitive impairment and dementia related tests. Genetic testing (*C9orf72*, *GRN*, and *MAPT*) was not recommended if there was no family history of frontotemporal dementia. In addition, other factors were considered including the cost factor, patient preference, lack of utility and lack of genetic counseling management if positive.

FTD clinical subtypes were classified into the standard behavioral variant, semantic aphasia, non-fluent aphasia, cortico-basal degeneration variant, progressive supranuclear palsy variant, FTD and amyotrophic sclerosis variant in accordance with currently accepted classification (36). Etiological entities were based on clinical history, cognitive, behavioral, laboratory and imaging analyses and categorized as:

1. Traumatic Brain Injury: Centers for Disease Control and ICD-10 criteria for mild and moderate TBI (37).
2. Frontotemporal lobe degenerations and dementias: Daphne Screening test, FBI test (if the score is ≥27).
3. Vascular Cognitive Disorder and dementia: AHA/ASA criteria (38).
4. Neurotoxicological: Gulf War Illness (GWI), Agent Orange, Camp Lejeune toxin exposure. For GWI Illness: Kansas, Haley or Institute of Medicine criteria (9, 39). In brief, there needed to be least 3 of the 6 symptom domains positive (chronic fatigue, cognitive disorders/headache/mood disorder, dyssomnia, somatic pain, gastrointestinal symptoms, typically chronic diarrhea, respiratory symptoms and skin rashes) for a GWI diagnosis to be made in the context of having been deployed in the 1991 Desert Storm conflict. For Agent Orange and Camp Lejeune toxin exposure, appropriate historical time frame and geographic association was used.
5. Alzheimer’s dementia: NIH Makhann criteria (40) with specific attention to subtype variants of frontal Alzheimer’s, amnestic, visuospatial, logopenic progressive aphasia and posterior cortical atrophy syndrome (Benson’s syndrome).
6. Lewy Body dementia: McKeith criteria (41).

The overall clinical and investigative approach in the study was the delineation of FTD into 3 principal categories, namely by FTD clinical variants, by etiological subtypes, and by a number of common and less common subsyndromes.

### Statistical analyses

Baseline, demographic, cardiovascular risk factors, cognitive, behavioral data, neuroimaging and presumed etiological categories, were compared between groups. Analysis of variance (ANOVA) and chi-square tests (for categorical variables) were used to examine whether the means between our groups (TBI, vascular, GWI, and other) were statistically different. In addition, a multivariate linear regression analysis was used to examine factors associated with FRSBE scores. A significance level of *p*-values below 0.05 was chosen. Statistical analysis was performed using Stata Version 16 MP (StataCorp, College Station, TX, USA) for data management and data analysis. Dispersion and position indices were depicted by interquartile ranges (25th, 50th, 75th).

## Results

**Figure 2:**
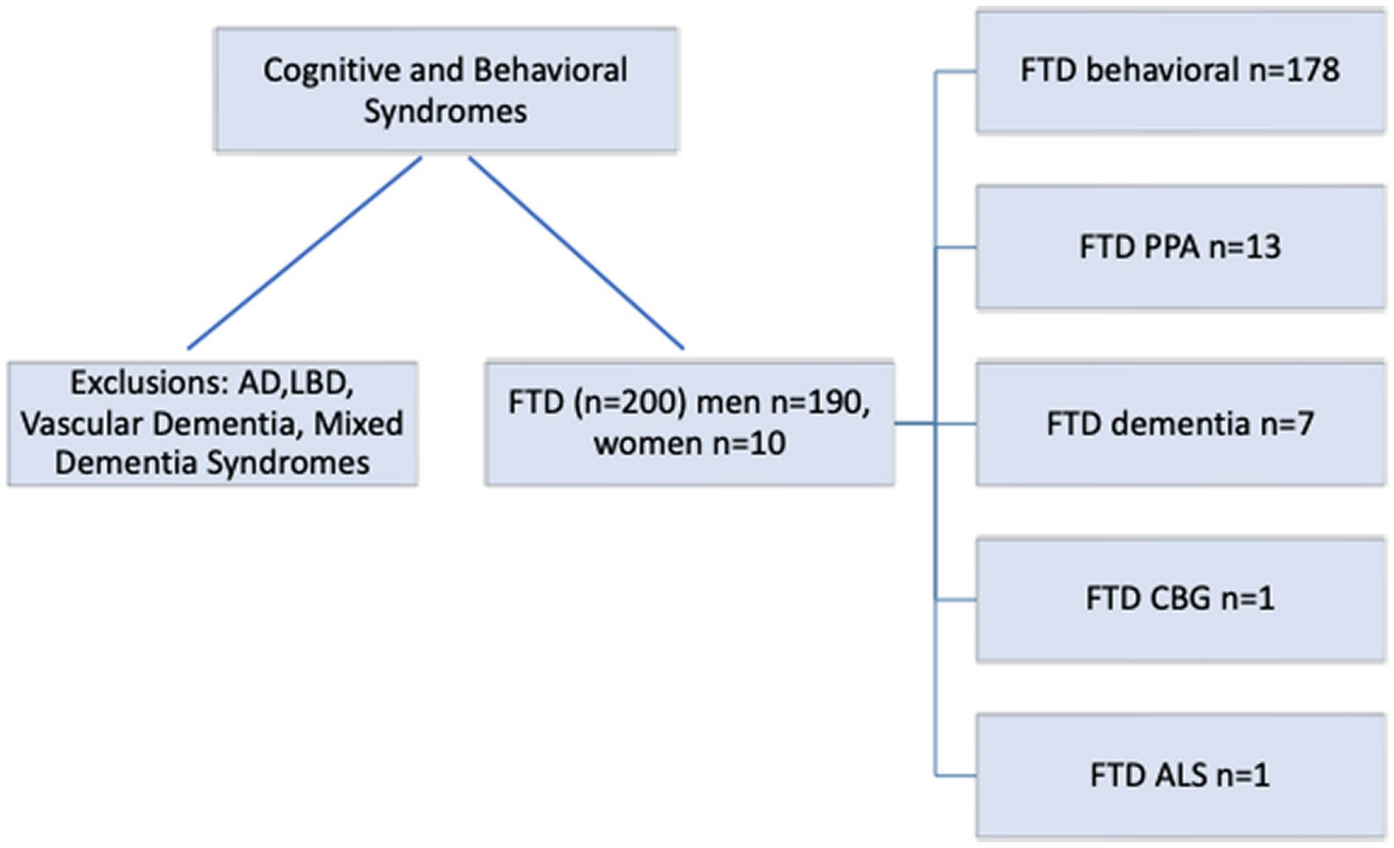
Frontotemporal disorders (FTD) cascade and variants. AD, Alzheimer’s Disease; LBD, Lewy Body Dementia; FTS, Frontotemporal Syndrome; CTE, Chronic Traumatic Encephalopathy.

Of the 200 patients with FTD, there were 190 men and 10 women with a mean age of 59.6 years (range 27–90 years) and mean education years of 13.8 (range 8–20 years). Screening cognitive testing with the MOCA tool and frontotemporal behavioral screening with the Daphne test was possible in all patients. This yielded an overall frequency of the generally accepted FTD clinical subtypes in this population, as noted in Figure 2.

**Figure 3:**
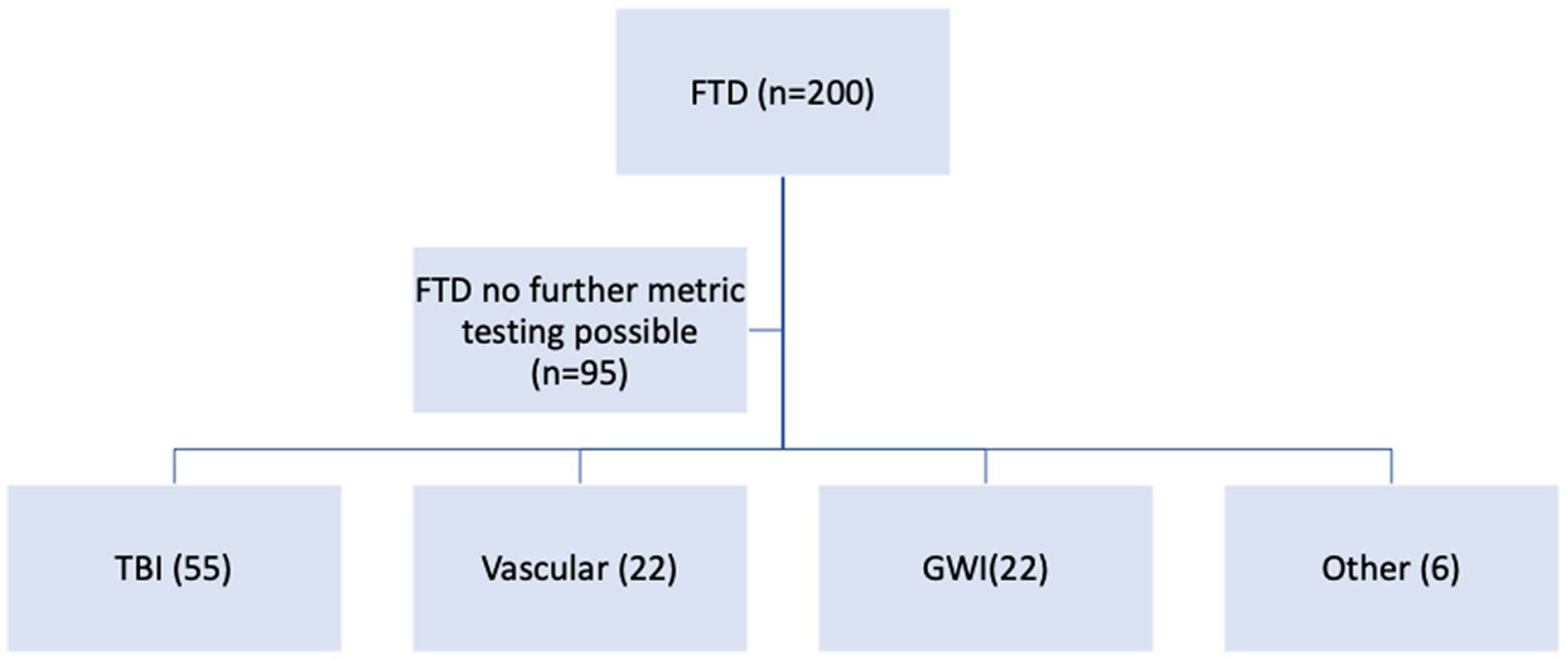
FTD syndrome metric analysis and etiological categories (*n* = 105).

**Figure 4:**
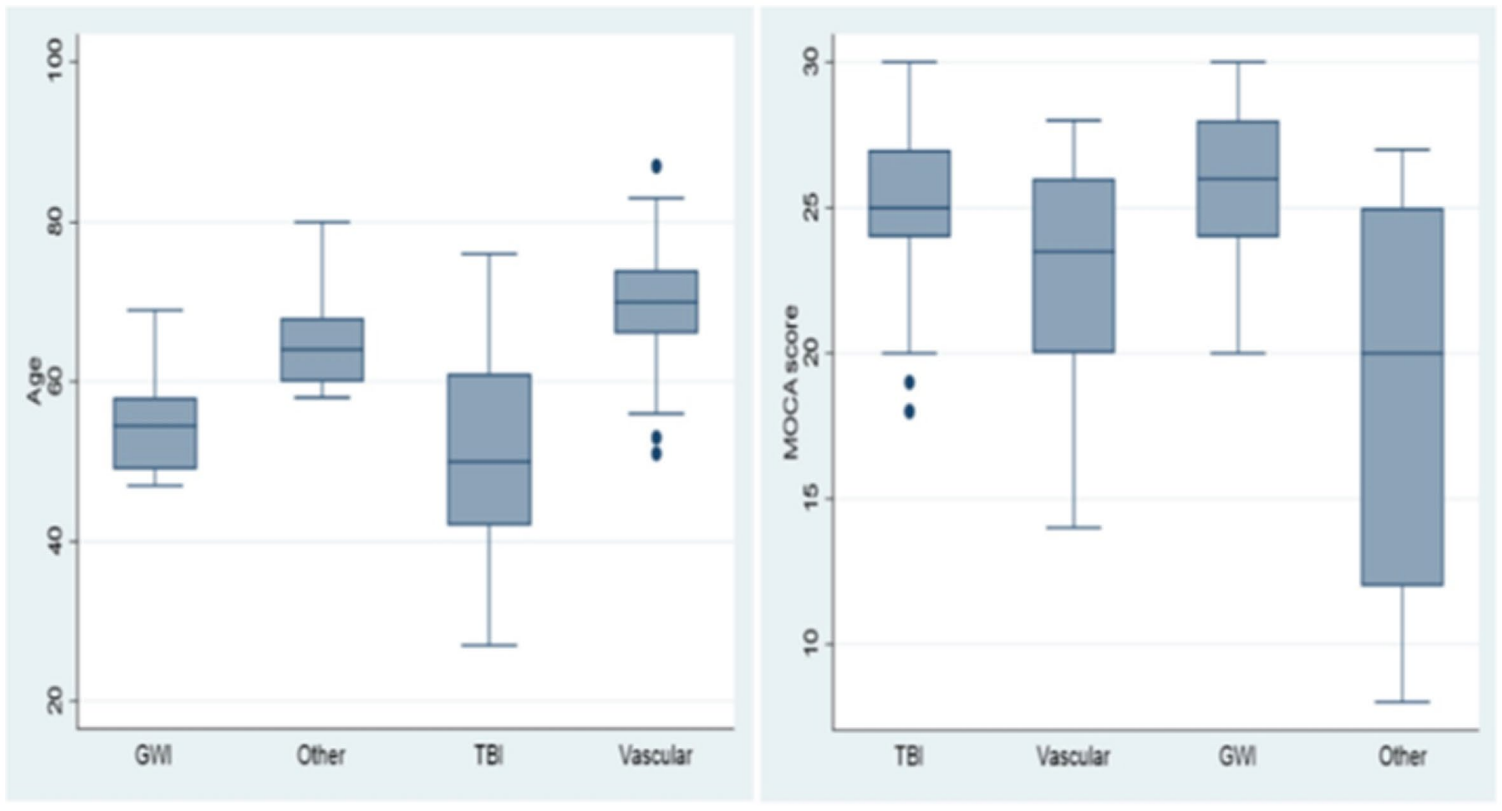
Age and MOCA scores, box and whisker plots. Age and MOCA scores differed significantly in the TBI and GWI groups.

Further behavioral neurological analysis with standardized further metric behavioral neurological evaluation was possible in only 105 of the 200 patients. This was primarily due to inability to perform the tests, lack of follow up, missing data, significant comorbidities or medication related effects hindering reliable testing. Analysis of the etiological groups (Figure 3) revealed significantly different younger age of the TBI and GWI veterans who also had higher MOCA scores, the latter which were overall borderline normal. Both the TBI and GWI groups had significantly different higher MOCA scores compared to the “older” vascular group (Figure 4). The TBI group also had significantly more abnormalities of hypometabolism, noted on the PET brain scans. Table 1 summarizes the means, standard deviations, and *p*-values from the statistical tests performed for the four different groups. Analysis of the behavioral neurological testing was notable for the findings that once frontotemporal disorder had been diagnosed by the Daphne (or Rascovsky) criteria (42), the four different etiological groups consistently had abnormal FRSBE scores for the 3 principal frontal presentations of (i) abulia/apathy, (ii) disinhibition, and (iii) executive dysfunction as well as abnormal FBI scores. Importantly there was no significant difference amongst the etiological groups (Table 1). This may be regarded as a significant finding underscoring the diagnostic accuracy of the initial FTD diagnosis all of which had similar deficits in the three principal frontal behavioral deficits, namely abulia/apathy, disinhibition and executive dysfunction.

**Table 1:** Comparison among different sub types of FTD.

Item	Subtype				*p*-value
	TBI	Vascular	GWI	Other	
No. of cases	*N* = 55	*N* = 22	*N* = 22	*N* = 6	
Age, years	51 (12.86)	69.5 (9.19)	54.59 (6.37)	65.67 (7.87)	<0.001
Gender, n (%)					0.101
Female	4 (7.27)	1 (4.55)	1 (4.55)	2 (33.33)	
Male	51 (92.73)	21 (95.45)	21 (95.45)	4 (66.67)	
Education, years	13.98 (2.21)	13.5 (2.26)	14.59 (2.17)	14.17 (2.71)	0.451
MOCA score	25.24 (2.65)	23.23 (3.56)	25.45 (3.31)	18.67 (7.47)	<0.001
FBI score	38.77 (11)	40 (11.84)	38.24 (11.1)	45.83 (9.11)	0.485
FRSBE_A	84.5 (20.68)	87.43 (16.99)	88.21 (18.46)	102.8 (22.99)	0.256
FRSBE_D	76.6 (19.51)	81.9 (23.31)	82.21 (18.28)	84.6 (20.67)	0.589
FRSBE_E	84.65 (13.81)	89.33 (17.97)	84.42 (14.14)	100 (19.03)	0.132
FRSBE_T	88.44 (16.71)	90.62 (28.11)	91.21 (15.16)	104.8 (21.05)	0.374
Bear_Fedio score	3.64 (4.62)	2.59 (4.7)	4.64 (4.39)	0.83 (2.04)	0.222
GGS, n (%)					0.080
No	28 (50.91)	16 (72.73)	9 (40.91)	5 (83.33)	
Yes	27 (49.09)	6 (27.27)	13 (59.09)	1 (16.67)	
KBS					<0.001
No	50 (90.9)	22 (100)	21 (95.5)	2 (33.33)	
Yes	5 (9.1)	0 (0)	1 (4.5)	4 (66.67)	
MRI brain scan					0.390
Normal	8 (14.5)	1 (4.5)	4 (18.18)	0 (0)	
Abnormal	47 (85.5)	21 (95.5)	18 (81.82)	6 (100)	
PET scan					0.147
Normal	34 (61.8)	8 (36.36)	13 (59.1)	2 (33.33)	
Abnormal	21 (38.2)	14 (63.64)	9 (40.9)	4 (6.67)	

All values are mean (SD) or otherwise count number (%) as indicated. ANOVA was performed for continuous variables and Pearson Chi-square test was conducted for categorical variables.

**Figure 5:**
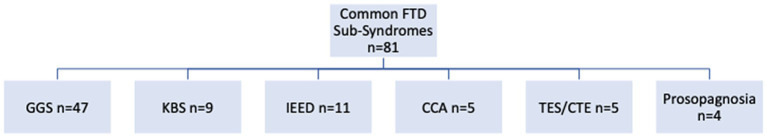
FTD clinically diagnosed sub-syndromes.

The most common subsyndromes associated with frontotemporal syndromes are depicted in Figure 5. Within this group, GGS was by far the most frequently delineated syndrome. Other relatively common syndromes included KBS, IEED, CCA, TES/CTE and prosopagnosia. Comparisons with the three principal frontal lobe syndrome clusters (abulia, disinhibition, executive dysfunction) revealed a significant association of abnormal disinhibition FRSBE T-scores with the GGS. The regression analysis supported the potential contribution of disinhibition behavior that related to this complex, relatively common behavioral syndrome in this series (Table 2). Linear regression models examining factors associated with FRSBE scores are presented in Table 2. Only patients with GGS had higher FRSBE-D and FRSBE-T scores. The other factors in our models did not have a statistically significant association with FRSBE scores.

**Table 2:** Linear regression model of the factors associated with FRSBE scores (*n* = 93).

	FRSBE-A score	FRSBE-D score	FRSBE-E score	FRSBE-T score
				
Patient gender				
Male	Reference	Reference	Reference	Reference
Female	2.56	0.46	−0.72	−0.19
	(−14.4–19.5)	(−16.5–17.4)	(−14.1–12.6)	(−17.3–17.0)
Education (years)	−1.67	−0.62	0.10	−0.77
	(−2.44–1.20)	(−2.44–1.20)	(−1.33–1.53)	(−2.60–1.07)
Age of patient	−0.14	0.28	0.20	0.13
	(−0.48–0.19)	(−0.05–0.62)	(−0.06–0.47)	(−0.20–0.47)
Geschwind-Gastaut	13.51	22.02**	9.50	17.62*
Syndrome diagnosis	(−2.64–29.66)	(5.87–38.17)	(−3.22–22.22)	(1.32–33.93)
Kluver body	11.62	4.15	5.20	8.96
Syndrome diagnosis	(−6.66–29.90)	(−14.13–22.43)	(−9.19–19.6)	(−9.50–27.42)
Bear fedio score	−1.47	−1.45	−1.03	−1.49
	(−3.29–0.35)	(−3.27–0.37)	(−2.46–0.40)	(−3.33–0.35)

95% confidence intervals are reported in parentheses. **p < 0.01, *p < 0.05.

Less common subsyndromes entities are depicted in Table 3. These syndromes, in particular, were notable, as they constituted the initial overriding, presenting symptoms and syndromes characterized into 16 separate conditions. They were conveniently subsumed under the 3 principal frontal syndromes of abulia, disinhibition and executive dysfunction and in addition the presumptive category of diaschisis related syndromes.

**Table 3:** Initial presenting syndromes of less common FTD subsyndromes.

Disinhibitory syndromes	
Field dependent behavior (imitation behavior, utilization behavior)	3
Hypersexuality including, gender dysphoria	3
Extreme happiness and jocularity	2
Hyperorality: eating about one gallon of ice cream per day	1
Lost fear of alligators as presentation (as part of Klüver Bucy Syndrome)	1
Hyperekplexia/startle reflex, new onset	1
*Diaschisis related*	
New artistic abilities (art or music)	3
Pan artistic ability (music, illustrative art, poetry, culinary, performing arts, oratorship, philosophy)	1
Stand-up comedian, increased literary skills, music skills, hypersexuality	1
Architectural brilliance	1
Hypervisual illusory spread syndrome	1
Continuous spontaneous sudden onset hyper-narration, post right middle cerebral artery stroke	1
Profound hypergraphia with compilation of 2 books of arbitrary notes, comments, presented	1
Excessive reading of the King James Bible (1,200 pages), 7 times at time of first visit	1
Profound new interest in astrophysics, loquacity and hypergraphia	1
*Abulia related*	
Diogenes syndrome (senile squalor syndrome)	1

## Discussion

The main findings of this retrospective analysis from the dedicated OVAMC cognitive neurological clinic included the frequency of FTD and the extensive array of behavioral neurological syndromes and sub-syndromes, embraced under the umbrella of FTD. Importantly, these presented mostly in the context of relatively milder cognitive impairment with the mean MOCA score in the TBI of 25.24 (SD 2.65) and GWI 25.45 (SD3.31), where 26 or greater is regarded as the normal range. In many tertiary medical centers, the MMSE and MOCA are screening tests that triage people with cognitive complaints depending on their scores into further investigations or no investigation at all. In this veteran population, the majority of people with TBI, had FTD, rarely in the dementia category because of relatively preserved basic ADLs and IADLs. Although frontotemporal syndromes may conjure up the more commonly known frontotemporal dementias, this study demonstrates that many of mild to moderate behavioral syndromes, in particular post TBI, post cerebrovascular and post neurotoxicological insults presented with frontotemporal disorders or syndromes and not dementias.

Several additional pertinent findings included the significantly different younger age of the TBI and GWI veterans who also had higher MOCA scores that were overall borderline normal. The MOCA score, the most commonly used cognitive screening study worldwide, being borderline normal in the TBI and GWI groups is important as many members in these groups would have missed being evaluated further in view of the normal MOCA scores, a relatively common practice. The study also presents a remarkable validation of the notable and extensive behavioral neurological repertoire of Andrew Kertesz’s “Banana Lady” exposition (43), wherein he tabulated 17 different frontal network syndromes and based the frontal behavioral inventory test on these findings. Herein we noted a veritable number of 16 differing frontal network syndromes aside from those evaluated by the Frontal Behavioral Inventory evaluation (*n* = 24). The more common sub-syndromes within the realm of FTD, delineated in this study, included GGS, KBS, DMIS, IEED and prosopagnosia. With regard to GGS, there was a trend toward a greater association with the TBI and GWI categories. At the time of writing, GGS have been reported in association with FTD in only two single case reports (44, 45). However, the remarkable frequency we detected in our retrospective series is noteworthy and warrants further attention as such syndromes are not only important for the person and family to understand, but have potential treatment and management options available. It may also be an important example of cerebral diaschisis with at times increased brain function and even superior or superlative brain functions developing particularly after TBI. Knowing more precise sub-syndromes and their etiologies enables the first step needed to deliver a precision medicine intervention.

From a pathophysiological point of view, the syndromes may be understood, with regards to the perspective of:

1. Anatomical – the particular frontotemporal lesions predilection consequent to TBI.
2. White matter fiber tract level disruption from the anterior temporal lobe to the inferior frontal lobe, the uncinate fasciculus.
3. Network level impairment: salience network.
4. Diaschisis, including subtypes of diaschisis at rest, functional, connectional and connectomal.

**Figure 6:**
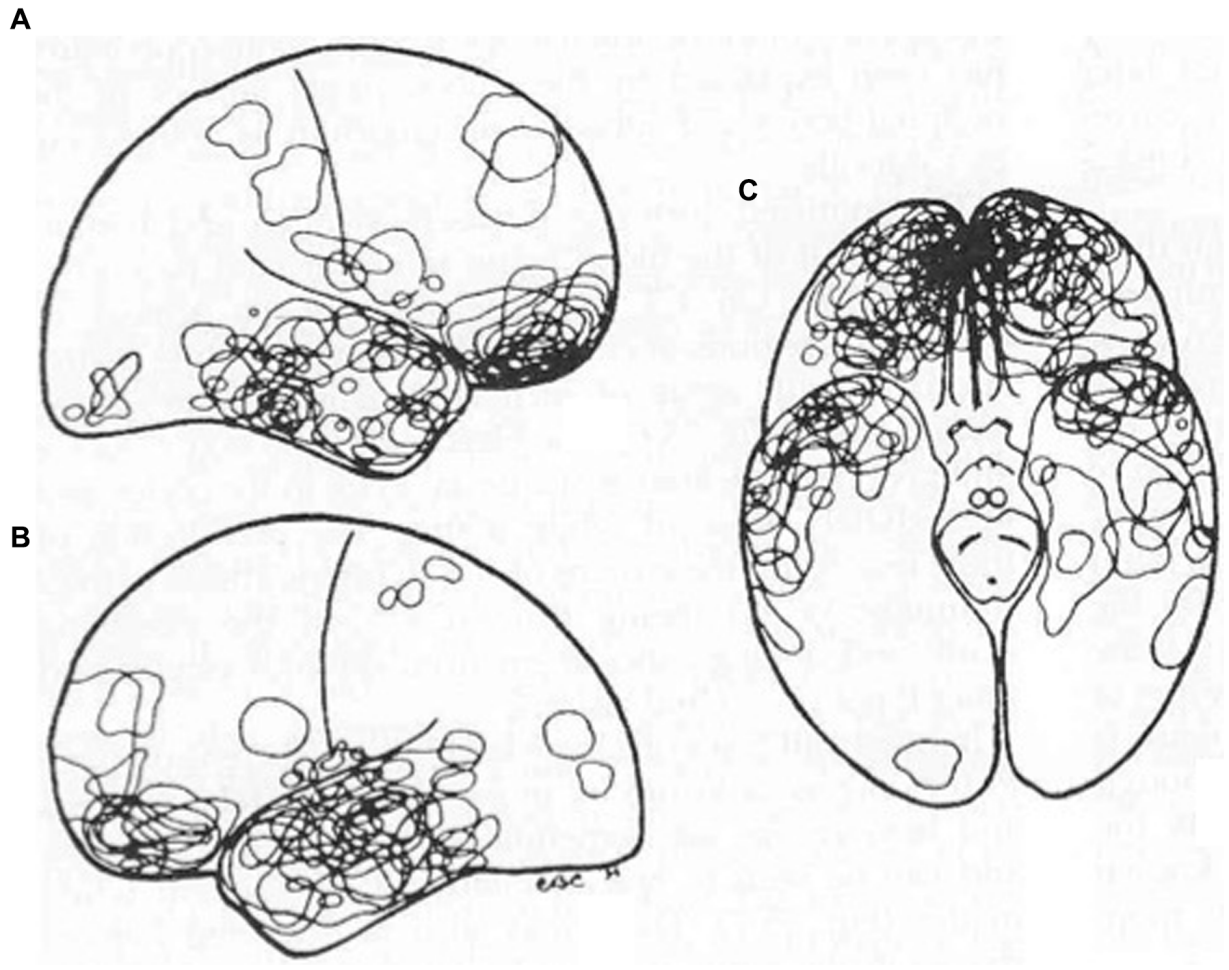
TBI: Fronto-temporal injury predilection distribution of contusions in 40 consecutive autopsy cases. Reproduced from Courville, Pathology of the central nervous system, Part 4 (46).

The particular predilection of inferior frontal and anterior temporal lobe injury post TBI is worth emphasis as depicted by neuropathological data (Figure 6) (46). This important pathological data has also been confirmed in the neurosurgical literature. Contusion indices in non-missile head injury (*n* = 151) revealed mean contusion indices (MCI) much more commonly in the frontal (MCI 5.7) and temporal lobes (MCI 5.4) as opposed to the sylvian fissure (MCI 2.7), occipital lobe (MCI1.2), parietal lobe (MCI 0.7) and cerebellum (MCI 0.9) (47).

With regards to GGS, the most common FTD sub-syndrome identified, the underlying abnormality may include: impairment of the anatomical circuit malfunction (uncinate fasciculus), a larger network dysfunction impacting the salience network, or secondary to a right hemisphere lesion, with diaschisis phenomena rendering the syndrome of GGS. The abnormal disinhibition FRSBE T-score association was interesting in that this might be a potential mechanism of diaschisis impacting the complex left temporal lobe cognitive and behavioral processes. An overarching insight premise relates to the extensive array of syndromes that are best explained by the brain network theory of remote injury (von Monokow) in both hodological and hodotopical effects of under-activation and overactivation (48). Brain network science (small world, rich club hubs) and the various hodological effects after lesions with clinically apparent diaschisis syndromes (at rest, functional, connectional) demand that the entire brain be evaluated no matter where the lesion topography (49). These can be accomplished by using metabolic positron emission tomography (PET) and resting state brain network imaging. Using such neuroimaging approaches this may further facilitate more precise diagnoses with more uniform subgroup identification. Furthermore, targeted treatments are then more likely to be successful as the specific brain area or network has been identified. The uncinate fasciculus matures the latest (during the 3rd and 4th decades) of all white matter fiber tracts (50). One may surmise that similar to other tertiary cortical circuitry this major tract may be the most vulnerable of all, to a panoply of neurological insults such as TBI, vascular and neurotoxicological injury. As it links the two of the most significant higher cortical function brain centers (frontal, temporal), the presentation of syndromes such as GGS would not be surprising. Furthermore, the study also illustrates, that, far from only focusing on neurological deficits, neurological hyperfunction in its myriad forms is equally informative and important for treatment prospects. Karl Deisseroth, one of the inventors of optogenetics notably proclaimed that the most accurate and insightful ways of deciphering the human brain is through language and the clinical interview, more so than sophisticated current neuroimaging modalities (51). At the present time, scored questionnaires such as the FRSBE, FBI and BFI are key tools that assist in deciphering these syndromes.

The salience network is a pivotal psychiatric network, linked to the to p-factor (psychopathology factor). The salience network is thought to play an important role, acting as a switch to deploy other major cerebral networks. Hence, lesions that affect this network, as an orchestrator influencing other networks, would have a disproportionate and at the same time may serve as a potential therapeutic target for neuromodulation devices, for example (52).

Behavioral and cognitive disorders are especially prone to failure in the constant stimulation and complicated nature that is part of the information age. This requires sustained attention, effective executive function and insightful reasoning for optimal decision-making, appropriate impulse control and inhibition of responses (53). The extensive networks and highly evolved cells, such as pyramidal, spindle and fork cells, that are characteristic of the association cortices, in particular, are targeted. No effective treatments currently exist, and the pharmaceutical industry has recently de-emphasized research in these areas. The reasons include the complexity, the uniquely significant primate association as opposed to the traditional rodent models, that have minimal association cortices. As FTD syndromes originate in the most plastic areas of the brain, these may also hold the promise of providing opportunities for future successful interventions (54). Recent wars have highlighted two signature syndromes that afflict a significant number of deployed personnel. Both affect brain regions with syndromes that are difficult to diagnose and treat. Mild and moderate traumatic brain injury (mTBI) and GWI fall within the domain of FTD (7, 39, 55). These constitute a panoply of disorders, where the presentation is predominantly behavioral, more so than with cognitive impairments. Decoding these syndromes and their causes, are pivotal to potentially effective treatments.

**Figure 7:**
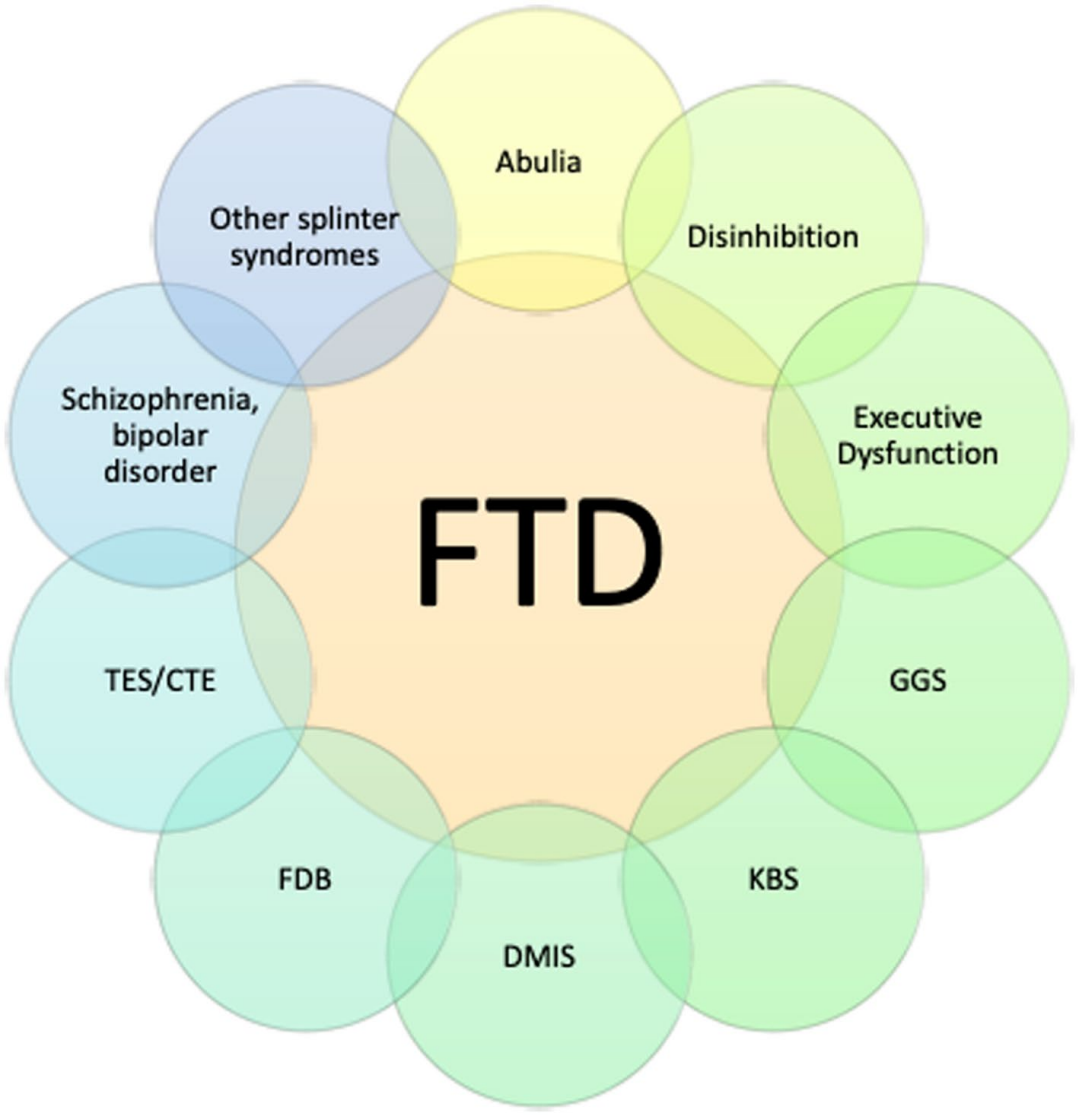
Frontotemporal disorders as a generic disorder encompassing many other associated syndromes. GGS - Geschwind-Gastaut syndrome, KBS - Klüver-Bucy syndrome, DMIS - delusional misidentification syndrome, FDB - field dependent behavior (imitation and utilization behavior), TES/CTE - traumatic encephalopathy/chronic encephalopathy syndrome.

How to make sense of this extensive panoply of syndrome? Frontotemporal syndromes can be viewed as a generic syndrome that encompasses and at times overlaps with several other neurological and neuropsychiatric conditions. Almost all can be subserved under the three principles frontal syndromes of abulia, disinhibition and executive dysfunction in various combinations (Figure 7). Importantly, FTD commonly co-occur with several others, both neurological and neuropsychiatric syndrome. The study also allows additional insights into these FTD syndrome complexes. For example, suicide rates were reported to be 56% higher in Veterans with TBI when compared to veterans without TBI. Suicide rates in US military veterans increased greater than 10-fold from 2006 to 2020 with presumed associations being more frequent mental health conditions, substance abuse, and firearm related violence gun violence (56). This study provides, perhaps an even more likely possibility. The predilection of TBI for the frontotemporal/uncinate fasciculus/salience network components harbor inhibitory circuitry, impulse control, as well as emotional regulation. A very plausible explanatory factor may related to the dysregulation of this important control circuitry which might explain the propensity for suicidal incidents.

### Potential limitations and strengths of the review

The most important shortcoming of this study includes the lack of comparison group. Data collection was often incomplete and there was susceptibility to selection and measurement bias. Another limitation pertains to the imprecise assessment of both PTSD and migraine in this population and this data could not be reliably included. Migraine PTSD/depression/anxiety were commonly encountered in this population group. Although these syndromes were ubiquitous syndromes in the FTD population as a whole, more careful delineation amongst the subgroups were however not possible because of a wide variety of heterogeneous assessment scales. Reports from civilian TBI populations are known to be associated with PTSD in almost half the patients (57). The small number of women represented (*n* = 10) although all had the same comprehensive evaluations, does not allow further analysis of gender specific differences and is likely due to the predominance of combat related veterans in this analysis.

Scientific analysis may be regarded as having two basic approaches; the traditional hypothesis driven approach or one that uses a data driven approach. The former has a specific prediction based on a proposed hypothesis and is based on intuition. The data driven approach uses extensive data accrued which enables detection of specific patterns allowing a much more veritable hypothesis formulation. A major advantage of a case series, such as the present one, is that it can be regarded as a screening tool for the most plausible hypotheses that merit further investigation. Hence the strengths of this case series include; high external validity, a wide range of patients sample, the study was inexpensive, short in duration and no interference in the treatment process. A case series has specific advantages in generating new hypotheses and treatment efficacy and the external validity of a case series frequently exceeds that of a randomized controlled trial.

### Future recommendations: a precision medicine approach to cognitive and dementia care syndrome, etiology, and co-pathology

Increasingly a precision medicine approach is being heralded both from a syndrome and pathological treatment point of view. Recognizing multiple neuropathological entities in people with dementia improves understanding of diagnosis, prognosis, and expected outcomes from therapies (58). Rapidly accruing evidence is emerging for the efficacy of realm of lifestyle/behavioral interventions in most chronic neurological disease including cognitive impairment, Alzheimer’s disease, Parkinsons and more recently frontotemporal dementias (59–61). In addition, a surge of recent neuromodulatory device-based interventions such as magnetic and electrical brain stimulation devices, have shown promise for conditions such as Alzheimer’s, depression, schizophrenia and PTSD for example (62–65). However, a precision medicine type approach is first required to decipher the most likely underlying etiology, whether vascular compromise, biochemical deficiencies or neurotoxicological factors and eliminate these as a first step, if possible. For this reason, one of the objectives of this analysis was to pave the way for the establishment of a registry of people with FTD with deconstructing the FTD syndromes, similar to what Stephen Stahl, amongst others, have long proposed in neuropsychiatric disease (66). Once biochemical, toxins, infections and inflammatory abnormalities have been corrected wherever possible, the lifestyle/behavioral interventions can be initiated and monitored for success. The earliest neurobiological defect of cognitive disorders may well be at the neurovascular level with both clinical and neuroimaging studies supporting impaired cerebrovascular reactivity impairment as the first sign of compromise (67). This underscores the essential role of physical exercise which induces both generalized cardiovascular and cerebrovascular health as well as neurotrophic factors that lead to neurogenesis and augment brain circuitry (68). Physical exercise has particularly potent brain protective effects and has been shown to reduce the incidence of dementia by up to 50%. Healthy diet adherence, such as the various categories of keto type low carbohydrate, MIND diet and Mediterranean-type diet have consistently shown reduction in cardiovascular disease, cancer and dementia (69). Working memory may be regarded as the core frontal lobe function central to all other processes including attention, memory, executive function and inhibition, that improve with cognitive exercises that have been developed, such as Brain HQ and Cogmed computerized programs. Once optimization of lifestyle/behavioral factors has been attained, augmentation of the brain’s plasticity with neuromodulation (t-DCS, TMS, noninvasive vagal stimulation) devices may be implemented in an intervention group and a control group in groups of similar etiology, with greater chance of success. This has recently been confirmed in a pivotal study specifically for frontotemporal degenerations. FTD neurophysiological oscillatory signatures of gamma and theta to alpha wave coupling have been identified, opening the way for both pharmacological targets and neuromodulation interventions (70).

Frontotemporal syndromes, emanating from TBI and GWI pathophysiological processes, occur in the most sensitive and yet most plastic areas of the brain, with the frontal and anterior temporal lobes being preferentially vulnerable to degeneration during a person’s lifespan. However, the emerging concepts that the cerebral networks are impacted, as opposed to only focal lesions, are in support of lifestyle and vascular health promotion that can modify this aging process (71). The brain has tremendous neuroplasticity capability, having 4 x the plasticity of muscles, for example (72). An important recent study in FTD amelioration with physical exercise, emphasizes the efficacy and critical role of physical exercise (59). The accompanying editorial entitled that “diagnosis is not destiny” underscored the pivotal impact of physical exercise in people with FTD despite having potentially disadvantageous genotypes (73).

## Conclusion

By deconstructing FTD into the multiple subsyndromes and differing etiologies, this study may provide foundational insights, enabling a more targeted precision medicine approach for future studies, both in treating the sub-syndromes as well as the underlying etiological process.

## Data availability statement

The original contributions presented in the study are included in the article/supplementary material, further inquiries can be directed to the corresponding author.

## Ethics statement

The studies involving humans were approved by VHA Institutional Research Board Approval IRBNET ID: 1256151-4. Approval date: 04/11/2020. The studies were conducted in accordance with the local legislation and institutional requirements. The ethics committee/institutional review board waived the requirement of written informed consent for participation from the participants or the participants’ legal guardians/next of kin because retrospective analysis of existing data.

## Author contributions

MH: Conceptualization, Data curation, Investigation, Methodology, Resources, Supervision, Writing – original draft. FR: Data curation, Investigation, Writing – review & editing. LB: Investigation, Writing – review & editing. CK: Formal analysis, Software, Writing – review & editing.
